# Hospitalization mortality and associated risk factors in patients with polymyositis and dermatomyositis: A retrospective case-control study

**DOI:** 10.1371/journal.pone.0192491

**Published:** 2018-02-23

**Authors:** Chanyuan Wu, Qian Wang, Linrong He, Enhao Yang, Xiaofeng Zeng

**Affiliations:** 1 Department of Rheumatology, Peking Union Medical College Hospital, Peking Union Medical College & Chinese Academy of Medical Sciences, Beijing, China; 2 Department of Rheumatology, China-Japan Friendship Hospital, Chaoyang District, Beijing, China; National Institutes of Health, UNITED STATES

## Abstract

**Background:**

Polymyositis and dermatomyositis (PM/DM) are systemic autoimmune diseases with multiple organ involvements that manifest as muscular and cutaneous disorders, interstitial lung disease (ILD) and malignancies. However, information concerning the outcomes and associated factors for PM/DM patients who are hospitalized is limited.

**Methods:**

We retrospectively reviewed the medical charts of PM/DM patients admitted to a Chinese tertiary referral hospital (Peking Union Medical College Hospital, PUMCH) from 2008 to 2014. The deceased group included 63 patients who had “deceased discharge” status or were confirmed to have died within two weeks of hospital discharge. The demographic data, clinical manifestations, and direct causes of death were analyzed retrospectively. Medical records for 126 age- and sex-matched PM/DM patients were selected as controls from 982 inpatients successively admitted to the same center during the same period. In addition to the comparison of clinical manifestations between the two groups, binary logistic regression was conducted to explore the risk factors related to PM/DM mortality.

**Results:**

Over the past 6 years at PUMCH, the in-hospital mortality rate of PM/DM patients was 4.58%. The male gender and the elder patients had a high risk of death (P = 0.031 and P = 0.001 respectively). The three most frequent causes of death for PM/DM patients were pulmonary infection (35%), ILD exacerbation (21%) or both conditions (25%). Pulmonary infection (P<0.001, OR = 5.63, 95% CI, 2.37–13.36), pneumomediastinum (P = 0.041, OR = 11.02, 95%CI, 1.10–110.54), Gottron’s papules (P = 0.010, OR = 3.24, 95%CI, 1.32–7.97), and elevated erythrocyte sedimentation rate (ESR) (P = 0.005, OR = 9.9, 95%CI 2.0–49.0) were independent risk factors for in-hospital mortality of PM/DM patients.

**Conclusion:**

PM/DM patients continue to display high in-hospital mortality. Pulmonary infection is the strongest predictor of poor prognosis in PM/DM patients, followed by pneumomediastinum, Gottron’s papules, and elevated ESR.

## Introduction

Polymyositis and dermatomyositis (PM/DM) are idiopathic inflammatory myopathies characterized by subacute proximal limb weakness and inflammatory infiltration of skeletal muscles with or without distinctive skin rashes [1]. PM/DM is a rare disease that has a poor prognosis and high hospital mortality[2, 3]. Thus, an understanding of early clinical features associated with poor prognosis has important significance for clinical practice. Due to different case series compositions and study methods, there are also different results among previous studies. In addition to ILD and malignancy, previous studies revealed several other risk factors for poor PM/DM outcomes, including delayed diagnosis, infection, thrombocytopenia, antisynthetase antibody (ASA) positivity, heart involvement and diabetes [2–6]. In a study by Yu et al. [5] that analyzed 192 Chinese PM/DM cases from Taiwan, a high mortality rate (28.6%) was seen, and several factors associated with reduced survival periods for patients with PM and DM were identified, including thrombocytopenia, diabetes mellitus, ILD, and cancer. A recent study focusing on ASA-positive patients revealed that factors associated with survival include: extension of lung involvement, a low forced vital capacity (FVC) at baseline evaluation, and the presence of arthritis [7]. Most studies were the assessment of long-term survival, with few concerning to short-term mortality. The study of hospitalized mortality in America suggested that infection was the main cause of death[2], which was different from that of long-term mortality. But the understanding of the high-risk predictors and hospitalization mortality of PM/DM patients is still very limited, especially in China. In our study, we retrospectively reviewed the medical records of 982 PM/DM patients admitted to our center from 2008 to 2014 and performed a case-control analysis to identify possible related risk factors for death among PM/DM patients in China.

## Patients and methods

### Patients

With the approval of the Institutional Review Board (IRB) at Peking Union Medical College Hospital (PUMCH), we retrieved medical records for adult patients who were hospitalized at PUMCH with the diagnosis of “polymyositis (PM)” or “dermatomyositis (DM)” from October 2008 to October 2014. All patients were treated at PUMCH, a tertiary referral center for rheumatic diseases in China. Written informed consent from each patient was not obtained due to the retrospective nature of this study and the granted IRB approval. Study inclusion criteria were: 1) age>16 years-old and 2) fulfillment of Bohan and Peter criteria (1975) [8]. Also, among diagnoses considered as definite, probable, or possible PM/DM according to the number of criteria fulfilled (at least 4, 3, or 2, respectively, including dermatologic manifestations for DM diagnosis), patients with definite or probable PM/DM were included. Exclusion criteria were: 1) overlap syndromes with other connective tissue diseases, 2) clinically amyopathic dermatomyositis (CADM), 3) hospitalization for causes that were unrelated to myositis and its complications (such as pregnancy, cataract, appendicitis and nephrolithiasis with inactive PM/DM, 4) living status that cannot be confirmed by a “discharge against medical advice” and loss to follow up.

### Methods

Medical records for all patients were retrospectively reviewed. Data including demographic information, disease duration, clinical features, and complications at the time of patient admission, as well as causes of death were collected and analyzed. Among the study subjects, 63 patients (27 men, 36 women) died in hospital or within two weeks of hospital discharge. Medical charts of the deceased patients were independently reviewed by two senior rheumatologists. Medical records for 126 patients (54 men, 72 women) who survived during their hospital stay were selected using a systematic sampling method by matching age, sex, and disease subtype with deceased cases at a proportion of 1:2. Their clinical manifestations and treatments were then compared with that of the deceased group.

### Statistical analysis

Continuous variables were presented as mean ± standard deviation for normal distribution, or as median (quartiles) for skewed distribution. Categorical variables were presented as numbers and percentages. A Student’s t-test and Mann-Whitney U test were used to compare continuous variables, and a chi-square test and Fisher’s exact test were used to compare categorical data between the deceased and survival groups. A model of binary logistic regression was adopted to analyze the independent risk factors for death. Results from logistic regression were expressed as an odds ratio (OR) with 95% confidence interval (CI). A two-sided P value less than 0.05 was considered to be statistically significant. Analysis was performed with SPSS software (version 19.0, IBM SPSS, Chicago, USA).

## Results

A total of 982 patients discharged from PUMCH with a diagnosis of PM or DM between October 2008 and October 2014 were enrolled, including 63 patients who died during their hospital stay or within 2 weeks after hospital discharge. The in-hospital mortality rate (I-HMR) was 6.4%. Among the 63 deceased patients, 27 were male and 36 were female. The proportion of males who died was significantly higher than that seen for the surviving patients (42.9% vs. 29.9%, P = 0.031). The average age in the deceased group was 54.8 ± 14.5 years, which was significantly higher than that of the surviving patients (46.1 ± 16.4 years, P = 0.001). The three most frequent death causes for PM/DM patients were pulmonary infection (34.9%), ILD exacerbation (20.6%), or both (25.4%) (Table 1).

**Table 1:** Causes of death in PM/DM in-patients.

	n, (%)	Cases (n)	Proportion (%)
1	ILD exacerbation	13	20.6
2	Pulmonary infection	22	34.9
3	Pulmonary infection with ILD exacerbation	16	25.4
4	Pulmonary infection with heart failure	3	4.8
5	Infection with neutropenia	2	3.2
6	Cerebral hemorrhage due to anticoagulant	1	1.6
7	Gastrointestinal bleeding	1	1.6
8	Gastrointestinal perforation	1	1.6
9	Pulmonary artery hypertension	1	1.6
10	Lung cancer	1	1.6
11	Cardiopulmonary failure of unknown origin	2	3.2
	Total	63	100

ILD: interstitial lung disease.

In total, 63 deceased patients and 126 surviving patients were included in the case-control analysis. The deceased group presented more frequently with fever (61.9% vs. 37.3%, P = 0.001), mechanic’s hands (28.6% vs. 15.9%, P = 0.040), Gottron’s papules (73.0% vs. 45.2%, P<0.001), pulmonary hypertension (19.0% vs. 6.3%, P = 0.007), pneumomediastinum (14.3% vs. 0.8%, P<0.001) and pulmonary infection (68.3% vs. 27.8%, P<0.001) than the controls. Compared to the control group, the frequency of hypoalbuminemia (82.5% vs. 54.0%, P<0.001), thrombocytopenia (15.9% vs. 5.6%, P = 0.019) and elevated erythrocyte sedimentation rate (ESR) (79.4% vs. 42.9%, P<0.001) was higher in the deceased group (Table 2). Further analysis in anti Jo-1 negative patients indicated that Gottron’s papules (74.0% vs. 46.4%, P = o.oo3) and pneumomediastinum (14.0% vs. 0.9%, P<0.001) were still higher in the deceased group than the controls.

**Table 2:** Comparison of clinical manifestations between deceased and control PM/DM patients [x ± SD or n (%)].

	Deceased group (n = 63)	Control group (n = 126)	P value
Age (years-old)	53.29 ± 14.20	53.09 ± 13.93	0.79
Gender (M/F)	27/36	54/72	1.000
PM/DM	8/55	16/110	1.000
Course of disease (months)	5 (0.5,216)	3.8 (1,170)	0.76
Smoking	14 (22.2)	31 (24.6)	0.717
Myalgia	27 (42.9)	54 (42.9)	1.000
Muscle weakness	53 (84.1)	98 (77.8)	0.305
Fever	39 (61.9)	47 (37.3)	0.001
Heliotrope eruption	40 (63.5)	66 (52.4)	0.147
Heliotrope eruption in DM	40 (72.7)	66 (60.0)	0.108
Mechanic’s hands	18 (28.6)	20 (15.9)	0.040
Mechanic’s hands in DM	18 (32.7)	20 (18.2)	0.036
Gottron’s papules	46 (73.0)	57 (45.2)	< 0.001
Gottron’s papules in DM	46 (83.6)	57 (51.8)	< 0.001
Dysphagia	14 (22.2)	29 (23.0)	0.902
Hoarseness	9 (14.3)	16 (12.7)	0.761
Myocardial involvement	6 (9.5)	10 (7.9)	0.712
ILD	54 (85.7)	92 (73.0)	0.050
Pulmonary hypertension	12 (19.0)	8 (6.3)	0.007
Pneumomediastinum	9 (14.3)	1 (0.8)	< 0.001
Malignance	5 (7.9)	12 (9.5)	0.719
Pulmonary infection	43 (68.3)	35 (27.8)	< 0.001
Anti Jo-1 positive	13 (20.6)	14 (11.1)	0.192
Normal serum CK	25 (39.7)	46 (36.5)	0.671
Hypoalbuminemia	52 (82.5)	68 (54.0)	< 0.001
Thrombocytopenia	10 (15.9)	7 (5.6)	0.019
Lymphocytopenia	51 (81.0)	95 (75.4)	0.390
Elevated ESR	50 (79.4)	54 (42.9)	< 0.001
Corticosteroid pulse therapy during hospitalization	27 (42.9)	32 (25.4)	0.015

M/F: Male/Female. ILD: interstitial lung disease. CK: creatine kinase. ESR: erythrocyte sedimentation rate.

Univariate analysis showed that there were 11 factors associated with I-HMR at the level of P< 0.05. These included: fever, Gottron’s papules, mechanic’s hands, ILD, pulmonary hypertension, pneumomediastinum, thrombocytopenia, hypoalbuminemia, elevated ESR, pulmonary infection and corticosteroid pulse therapy during hospitalization. Furthermore, multivariate conditioned logistic regression analysis revealed that pulmonary infection, Gottron’s papules, pneumomediastinum and elevated ESR were independent risk factors for in-hospital mortality of PM/DM patients. (Table 3).

**Table 3:** Multivariate logistic regression analysis of risk factors for death of PM/DM patients.

Factor	P value	OR value	95% CI
Pulmonary infection	< 0.001	5.63	2.37–13.36
Gottron’s papules	0.010	3.24	1.32–7.97
Elevated ESR	0.003	3.64	1.54–8.59
Pneumomediastinum	0.041	11.02	1.10–110.54

ESR: erythrocyte sedimentation rate.

## Discussion

Previous studies reported that the connective tissue disease PM/DM has a poor prognosis and high I-HMR. The reported 10-year survival rate ranged between 53% and 91% [4, 9–12]. A very recent population-based study from America reported a hospital mortality of 4.5% [2]. However, data concerning I-HMR and correlative factors for PM/DM patients in China are limited. We aimed to address this gap in knowledge by retrospectively analyzing hospitalization data for 982 PM/DM patients admitted to PUMCH, a large tertiary medical center in China, between 2008 and 2014. In our study, the I-HMR of PM/DM patients was 6.4%, which was similar to that seen for a study conducted in America. The median disease course was 5 months in our patients’ cohort, so our focus was on short-term mortality which is much lower than previous studies of long-term mortality[12, 13]. Moreover, the risk factors associated with poor outcomes were also likely to different from these previous studies. Older male PM/DM patients presented with a poor prognosis in our cohort. Furthermore, logistic regression analysis revealed that pulmonary infection, Gottron’s papules, pneumomediastinum and higher ESR were independent risk factors for hospital mortality of PM/DM patients.

Infections, especially pulmonary infection, were the primary cause of death in PM/DM patients in China, which is similar to findings in other countries [10, 11, 14, 15]. In our study, pulmonary infection was the strongest predictor of hospital mortality among PM/DM patients (OR 5.63, CI 2.37–13.36). Murray et al. [2] noted that individuals with PM/DM were at increased risk of mortality from pneumonia compared to the general hospitalized population. Indeed, a high proportion of respiratory disorders, including ILD, pneumomediastinum, respiratory muscle weakness and esophageal dysfunction could increase the risk of infection in PM/DM patients [16]. A recent study from our center focusing on myositis patients in the ICU highlighted the extremely poor prognoses of patients with this condition, and concluded that the complex etiology of acute respiratory failure involving opportunistic infections and rapid progressive ILD distinguished idiopathic inflammatory myopathies (IIM) as a distinct entity as compared with other rheumatoid diseases [17]. In this study, 82.2% of patients in the deceased group had ILD, although this rate was not significantly different from that for the control group (P = 0.099) due to the high proportion of ILD among our control group (68.9%). According to our data, the rate of pneumomediastinum in the deceased group was significantly higher than that of the control group (P = 0.04, OR 11.2, CI 1.10–110.54). Notably, our data revealed that the deceased group had significantly higher (P = 0.015) rates of pulse steroid therapy than the control group during hospitalization, which is consistent with a report by Marie et al. [16] showing that use of immunosuppression and higher daily doses of steroids increased susceptibility of PM/DM patients to infection. We agree that such types of therapy could indeed increase the risk of infection in PM/DM patients, and could have also reflected fundamentally more serious underlying disease activity in the deceased group. Moreover, our study suggested that the deceased group had a higher rate of hypoalbuminemia than the control group (P<0.001). Considering that hypoproteinemia in PM/DM patients was always due to poor nutritional status caused by dysphagia, persistent disease activity, and heavy consumption, all of these factors increased the risk of infection during hospitalization in PM/DM individuals.

Previous work reported that over 20% of PM/DM patients also had ILD, which is an important risk factor for the long-term prognosis of PM/DM [5, 18]. In our research, exacerbation of ILD and pulmonary infection with ILD exacerbation were both main causes of death (17.8% and 20%, respectively). As described above, our data for inpatients showed a very high proportion of ILD involvement (73.3%) in PM/DM patients. This rate was higher than other PM/DM cohorts, as well as relative to data for inpatients at other hospitals in China. This apparent selection/choice bias for high ILD involvement in our study could be, at least in part, responsible for the result that no correlation was found between ILD and the deceased group. On the other hand, we did not distinguish between different types of ILD, although rapidly progressive ILD could be associated with hospitalization death of PM/DM, a possibility that was verified in our previous ICU data analysis [17]. More prospective studies on this association are needed.

Myocardial involvement in PM/DM is not common, but is often suggested to be a risk factor for poor prognosis [3]. The incidence of myocardial involvement was reported to range between 9% and 72%, with most cardiac lesions being subclinical. Heart failure and arrhythmia are common clinical manifestations of myocardial involvement [19]. However, our study revealed no association between myocardial involvement and a high in-hospital mortality for PM/DM patients. Clinically, the occurrence of myocardial involvement and the elevation of serum myocardial enzymes were not in parallel in PM/DM patients [20]. Moreover, serum markers for early diagnosis and evaluation of myocardial involvement remain unreliable, whereas echocardiography and electrocardiogram also lack sufficient sensitivity [21]. In light of these limitations, cardiac magnetic resonance imaging (MRI) may be a useful method for early diagnosis of myocardial involvement in PM/DM patients.

Malignancies are regarded as a severe complication in PM/DM individuals and are associated with prognosis. Earlier studies reported that incidence of malignancy was between 3 and 30% [13, 22, 23]. Our cohort had 17 cases of malignancy, including 5 in the deceased group (7.9%) and 12 in the control (9.5%). Although most previous studies concerning long-term prognosis indicated that malignancy is major determinant of PM/DM [6, 22, 24], we saw no significant difference (P = 0.719) between the two groups in this study. As we mentioned before, although the patients died after discharge (in two weeks) were included, our main concern was still the short-term mortality. This difference may be due to distinction of in-hospital mortality and long term mortality, or the sample number and follow-up time.

Additionally, our study demonstrated that elevated ESR (OR 9.9, CI 2.0–49.0) and Gottron’s papules (OR 13.2, CI 2.6–67.0) were individual risk factors for hospitalization mortality in PM/DM patients. Previous reports demonstrated that elevated ESR was a poor prognostic indicator for PM/DM [25, 26]. Furthermore, some studies reported that elevated ESR was a risk factor for the occurrence of malignancies, which were associated with the long-term prognosis of PM/DM patients [27], although information concerning the relationship between ESR and hospital mortality of PM/DM patients remains limited. Regarding other risk factors, Gottron’s sign is regarded as a poor prognosis factor for PM/DM patients complicated with ILD [28], whereas a study by Fiorentino et al. indicated that Gottron’s sign (rather than papules) is associated with anti-MDA5 antibodies, which might be relevant to the rapid progression of ILD [29]. We have analyzed anti Jo-1 negative patients and indicated that Gottron’s papules and pneumomediastinum were higher in the deceased group than the controls. We supposed there may be a certain amount of anti MDA5 positive patients who could be marked by Gottron’s papules and/or pneumomediastinum. Unfortunately, the data for our retrospective study could not provide more supporting evidence for the relationship between these factors.

In summary, pulmonary infection, pneumomediastinum, Gottron’s papules and elevated ESR were independent risk factors for hospitalization mortality of PM/DM patients in China. Moreover, in our study, pulmonary infection was the strongest predictor of poor prognosis in PM/DM patients.

## Supporting information

S1 Table Primary data of deceased and control PM/DM patients. (XLS)
